# Association of Electronic Health Record Use With Physician Fatigue and Efficiency

**DOI:** 10.1001/jamanetworkopen.2020.7385

**Published:** 2020-06-09

**Authors:** Saif Khairat, Cameron Coleman, Paige Ottmar, Dipika Irene Jayachander, Thomas Bice, Shannon S. Carson

**Affiliations:** 1 Carolina Health Informatics Program, University of North Carolina at Chapel Hill; 2 School of Nursing, University of North Carolina at Chapel Hill; 3 Department of Preventive Medicine, University of North Carolina at Chapel Hill; 4 Gilling’s School of Public Health, University of North Carolina at Chapel Hill; 5 Pulmonary Diseases and Critical Care Medicine, University of North Carolina at Chapel Hill

## Abstract

**Importance:**

The use of electronic health records (EHRs) is directly associated with physician burnout. An underlying factor associated with burnout may be EHR-related fatigue owing to insufficient user-centered interface design and suboptimal usability.

**Objective:**

To examine the association between EHR use and fatigue, as measured by pupillometry, and efficiency, as measured by mouse clicks, time, and number of EHR screens, among intensive care unit (ICU) physicians completing a simulation activity in a prominent EHR.

**Design, Setting, and Participants:**

A cross-sectional, simulation-based EHR usability assessment of a leading EHR system was conducted from March 20 to April 5, 2018, among 25 ICU physicians and physician trainees at a southeastern US academic medical center. Participants completed 4 simulation patient cases in the EHR that involved information retrieval and task execution while wearing eye-tracking glasses. Fatigue was quantified through continuous eye pupil data; EHR efficiency was characterized through task completion time, mouse clicks, and EHR screen visits. Data were analyzed from June 1, 2018, to August 31, 2019.

**Main Outcomes and Measures:**

Primary outcomes were physician fatigue, measured by pupillometry (with lower scores indicating greater fatigue), and EHR efficiency, measured by task completion times, number of mouse clicks, and number of screens visited during EHR simulation.

**Results:**

The 25 ICU physicians (13 women; mean [SD] age, 32.1 [6.1] years) who completed a simulation exercise involving 4 patient cases (mean [SD] completion time, 34:43 [11:41] minutes) recorded a total of 14 hours and 27 minutes of EHR activity. All physician participants experienced physiological fatigue at least once during the exercise, and 20 of 25 participants (80%) experienced physiological fatigue within the first 22 minutes of EHR use. Physicians who experienced EHR-related fatigue in 1 patient case were less efficient in the subsequent patient case, as demonstrated by longer task completion times (*r* = −0.521; *P* = .007), higher numbers of mouse clicks (*r* = −0.562; *P* = .003), and more EHR screen visits (*r* = −0.486; *P* = .01).

**Conclusions and Relevance:**

This study reports high rates of fatigue among ICU physicians during short periods of EHR simulation, which were negatively associated with EHR efficiency and included a carryover association across patient cases. More research is needed to investigate the underlying causes of EHR-associated fatigue, to support user-centered EHR design, and to inform safe EHR use policies and guidelines.

## Key Points

**Question:**

What is the association between electronic health record use and physician fatigue and efficiency?

**Findings:**

In this cross-sectional study of 25 physicians completing 4 simulated cases of intensive care unit patients in the electronic health record, all physicians experienced fatigue at least once and 80% experienced fatigue within the first 22 minutes of electronic health record use, which was associated with less efficient electronic health record use (more time, more clicks, and more screens) on the subsequent patient case.

**Meaning:**

Physicians experience electronic health record–related fatigue in short periods of continuous electronic health record use, which may be associated with inefficient and suboptimal electronic health record use.

## Teaser

This cross-sectional study examines the association between electronic health record use and fatigue and efficiency among intensive care unit physicians completing a simulation activity in a prominent electronic health record.

## Introduction

Use of electronic health records (EHRs) is directly associated with physician burnout.^1,2^ Many physicians have voiced dissatisfaction with the click-heavy, data-busy interfaces of existing EHRs.^1,3^ Other factors associated with EHR frustration include scrolling through pages of notes and navigating through multiscreen workflows in the search for information.^4^ Excess EHR screen time leads to emotional distress in physicians and limits face-to-face contact with patients, resulting in higher rates of medical errors.^5,6^ Thus, common attitudes among physicians toward the EHR include “inefficient,”^7^ “time-consuming,”^8^ and “exhausting.”^9^

Patient safety and quality of care depend on EHR usability.^6,10^ This fact is especially true in intensive care units (ICUs), where critically ill patients generate, on average, more than 1200 individual data points each day,^11^ and it has been estimated that ICU clinicians monitor about 187 alerts per patient per day,^12^ mostly through the EHR. Poor EHR design exacerbates this cycle, potentially affecting decision-making and causing delays in care,^6^ medical errors,^6,13^ and unanticipated patient safety events, especially in high-risk environments.^14,15,16^ Despite the challenges of today’s EHR interfaces, much work remains to achieve truly user-centered EHR systems with better designs that improve efficiency (ie, mouse clicks and time), streamline decision-making processes, and support patient safety.^17,18^ Whereas traditional EHR usability testing often focuses on intrinsic, vendor-specific aspects of the system (such as screen layouts and workflows), it is important to distinguish EHR efficiency as extrinsic and dynamic—as much a function of the user as the system itself.

Eye tracking, the study of movements of the eyes, and pupillometry, the measurement of pupil dilation, have been applied in many nonclinical domains. Eye-tracking research, which typically analyzes fixation duration, gaze points, and fixation counts,^19^ has been used to investigate users’ engagement with advanced interfaces and website design, as well as visual attention in video games.^20,21,22^ In biomedicine, eye-tracking techniques have mostly been used to understand factors associated with interpretation of radiology studies, identification of medication allergies, reading progress notes in the EHR, and physician attention during cardiopulmonary bypass.^23,24,25,26^

Pupillometry, however, remains underused in medical research despite its promising capabilities. The degree of pupillary constriction during a task is a validated biomarker for fatigue and alertness.^27,28^ Research has consistently shown that during conditions of fatigue, baseline pupil diameters are smaller than normal.^29,30,31,32,33^ Reduction in pupil size by 1 mm has been associated with signs of tiredness.^29^ Change in pupil diameters is typically small, ranging between 0.87 and 1.79 mm from normal pupil size.^29^ In 1 study, significant correlations were found between individual differences in pupil size and mental workload for patients with anxiety, suggesting an association between these 2 indicators.^34^ Despite the potential of these technologies, eye tracking and pupillometry have yet to be used to understand EHR-related fatigue and its association with the user experience for clinicians.

The purpose of this study was to examine the association between EHR use and fatigue, as measured by pupillometry, and efficiency, as measured by completion time, mouse clicks, and number of EHR screens, among ICU physicians completing a simulation activity in a prominent EHR.

## Methods

We conducted a cross-sectional, simulation-based EHR usability assessment of a leading EHR system (Epic; Epic Systems) among ICU physicians and physician trainees at a southeastern US academic medical center, after approval from the University of North Carolina at Chapel Hill Institutional Review Board. Details of our study methods have been reported previously.^35^ Testing took place from March 20 to April 5, 2018. This study followed the Strengthening the Reporting of Observational Studies in Epidemiology (STROBE) reporting guideline.^36^ Participants provided written consent.

### Study Setting and Participants

The study was conducted at a southeastern US tertiary academic medical center with a 30-bed medical ICU. We recruited participants through departmental emails and flyers. The eligibility criteria were: (1) medical ICU physicians (ie, faculty or trainee), (2) any previous experience using Epic in critical care settings, and (3) not wearing prescription glasses at the time of the study, to avoid interference with the eye-tracking glasses.

We recruited 25 medical ICU physicians for this study. Our sample exceeded the conventional usability study standards that recommend 5 to 15 participants to reveal 85% to 97% of usability issues.^37,38^ All testing took place in an onsite biobehavioral laboratory designed for simulation-based studies, equipped with a computer workstation with access to institutional EHR training environment (Epic Playground), away from the live clinical environment. The computer screen was the standard screen clinicians use in their practice setting, with appropriate ergonomic placement, ambient lighting, and seating. Participants were recruited for a 1-hour individual session. Prior to each session, the principal investigator (S.K.) explained the study protocol to participants, assuring them that our study aim was to assess EHR efficiency rather than their clinical knowledge.

We asked participants to wear eye-tracking glasses (Tobii Pro Glasses 2; Tobii AB; eFigure 1 in the Supplement), which are extremely lightweight and do not impair vision. On sitting at the work station, the glasses were calibrated for each participant to establish individual baseline pupil size. Each participant then logged into the EHR training environment and completed, in sequence, the same 4 ICU patient cases, which were developed by a domain expert (T.B.) and physician trainee (C.C.), as published previously.^35^ Participants were asked to review a patient case (eTable 1 in the Supplement) and notify the research assistant when they completed their review. At that point, the research assistant asked the participant a series of interactive questions that involved verbal responses as well as completing EHR-based tasks. There were 21 total questions and tasks across the 4 patient cases (eTable 1 in the Supplement). Pupil diameter was recorded continuously during the entire study, and all participants used the same eye-tracking glasses. After participants completed the 4 cases, they removed the eye-tracking glasses, indicating the end of the study. Each participant received a $100 gift card on completion.

### Outcomes

Primary outcomes were physician fatigue, measured by pupillometry (with lower scores indicating greater fatigue), and EHR efficiency, measured by completion time, number of mouse clicks, and number of screens visited during EHR simulation.

### Measurements

#### Quantification of Fatigue

Fatigue was measured on a scale from −1 to 1, as advised by an eye-tracking specialist, with lower scores than baseline indicating signs of fatigue, and negative scores (between 0 and −1) indicating actual physiological fatigue. Simulation sessions occurred across a mix of conditions (morning and afternoon), with some participants undergoing testing on a day off or nonclinical day and other participants coming from a clinical shift in the medical ICU. Thus, to account for individual differences in baseline pupil size, we calculated a baseline for each participant, defined as the participant’s mean pupil size for the first 5 seconds during calibration. We then determined acute changes in pupil size during the simulation exercise by subtracting each participant’s baseline pupil size from his or her pupil size for each question or case. For each participant, we analyzed changes in pupil size to generate fatigue scores associated with the EHR simulation exercise by question and by case, according to the equations:

Fatigue per question:
  Left or Right Eye Fatigue Score = (Mean of Pupil Size During Last 5 Seconds of Answering a Given Question) − (Mean of Pupil Size During First 5 Seconds of Asking a Given Question).
Fatigue per case:
  Left or Right Eye Fatigue Score = (Mean of Pupil Size During Last 5 Seconds of the Case) − (Mean of Pupil Size During First 5 Seconds of Entire Case).
Total Fatigue Score = [(Right Eye Fatigue Score) + (Left Eye Fatigue Score)]/2.

#### Quantification of EHR Efficiency

We measured EHR efficiency by using standard usability software that ran in the background during the simulation exercises (TURF; University of Texas Health Science Center). This software includes a toolkit to capture task completion time, number of mouse clicks, and number of visited EHR screens for each case.

### Statistical Analysis

Data were analyzed from June 1, 2018, to August 31, 2019. We calculated summary and descriptive statistics for the primary outcome measures of fatigue and EHR efficiency, including subgroup analysis by sex and clinical role. To explore the association between fatigue and efficiency, we calculated Pearson correlation coefficients between fatigue scores and the EHR efficiency measures (time, mouse clicks, number of EHR screens visited). All analysis was performed in SPSS, version 22.0 (SPSS Inc). All *P* values were from 2-sided tests and results were deemed statistically significant at *P* < .05.

## Results

We recorded a total of 14 hours and 27 minutes of EHR activity across 25 ICU physicians (13 women; mean [SD] age, 32.1 [6.1] years) who completed a simulation exercise involving 4 patient cases (mean [SD] completion time, 34:43 [11:41] minutes) (Table). There was an uneven distribution by clinical role, with more resident physicians (n = 11) and fellows (n = 9) than attending physicians (n = 5). Mean (SD) age tended to mirror clinical role, with residents being the youngest group (29.0 [1.4] years; fellows, 32.7 [0.5] years; and attending physicians, 44.0 [6.5] years). An inverse trend was noted between clinical role and the mean (SD) self-reported time spent per week using the EHR, with residents spending the most time (41.2 [13.5] hours) and attending physicians spending the least (8.3 [7.2] hours). The mean self-reported years’ experience with Epic was similar across all 3 clinical roles.

**Table.** Study Participant Demographic Characteristics, Descriptive Variables, and EHR Efficiency Variables

Variable	No.	Age, mean (SD), y	Experience with EHR system, mean (SD), y^a^	Time using EHR system, mean (SD), h/wk^a^	Case fatigue scores, median (range)^b^				EHR efficiency, mean (SD)		
					Case 1: multiorgan failure	Case 2: respiratory failure	Case 3: severe sepsis	Case 4: volume overload	Time, min:s	Mouse clicks, No.	EHR screens, No.
Total	25	32.1 (6.1)	3.9 (1.3)	35.1 (21.4)	0.075 (−0.183 to 0.409)	−0.015 (−0.804 to 0.634)^c^	−0.033 (−0.789 to 0.260)^c^	−0.030 (−0.464 to 0.801)^c^	34:43 (11:41)	304 (79)	85 (19)
Clinical role											
Resident	11	29.0 (1.4)	4.0 (0.4)	41.2 (13.5)	0.016 (−0.183 to 0.231)	−0.122 (−0.804 to 0.223)^c^	−0.087 (−0.789 to 0.260)^c^	−0.076 (−0.464 to 0.801)^c^	36:54 (14:43)	411.6 (90)	94 (21)
Fellow	9	32.7 (0.5)	5.7 (0.9)	39.3 (22.2)	0.053 (−0.103 to 0.409)	0.002 (−0.267 to 0.634)	−0.030 (−0.223 to 0.173)^c^	0.001 (−0.226 to 0.207)	28:51 (05:52)	312.7 (88)	81 (16)
Attending physician	5	44.0 (6.5)	3.8 (0.4)	8.3 (7.2)	0.091 (−0.141 to 0.227)	0.132 (−0.04 to 0.247)	0.100 (−0.074 to 0.145)	0.007 (−0.178 to 0.361)	40:28 (06:10)	316.8 (71)	73 (8)
Sex											
Female	13	29.2 (0.7)	3.5 (0.8)	50.0 (22.2)	0.043 (−0.156 to −0.061)	−0.024 (−0.267 to 0.061)^c^	−0.043 (−0.314 to −0.054)^c^	0.001 (−0.225 to 0.600)	31:37 (08:22)	355 (101)	89 (26)
Male	12	32.3 (2.0)	4.6 (3.5)	19.8 (12.1)	0.086 (−0.183 to 0.225)	0.067 (−0.267 to 0.061)	−0.052 (−0.789 to −0.616)^c^	−0.088 (−0.463 to −0.102)^c^	38:04 (13:40)	301 (66)	87 (15)

Abbreviation: EHR, electronic health record.

^a^ Self-reported variables.

^b^ Score range, –1 to 1; lower scores indicate more fatigue; higher scores indicate less fatigue.

^c^ Negative scores (<0.0) suggest actual physiological fatigue.

### Physician Fatigue

All participants experienced actual physiological fatigue at least once throughout the EHR simulation exercise, as evidenced by a negative fatigue score. Total fatigue scores for participants ranged from −0.804 to 0.801 (eTable 2 in the Supplement).

**Figure 1.**
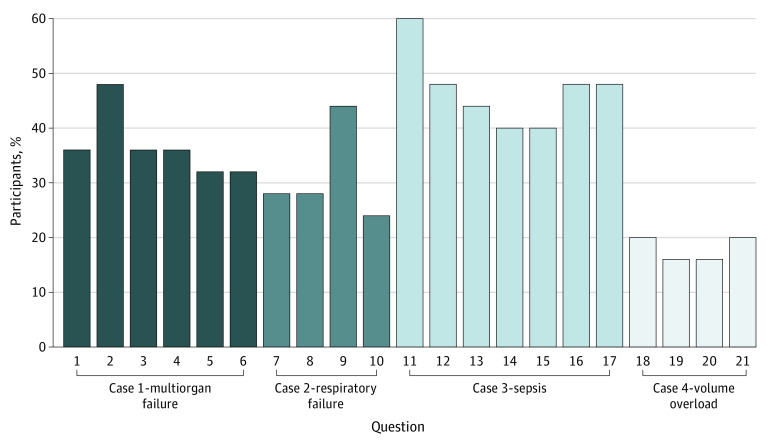
Percentage of Participants (N = 25) Experiencing Fatigue by Question See eTable 1 in the Supplement for description of tasks and questions.

Fatigue scores varied by case and by question or task. Figure 1 shows the distribution of physicians experiencing fatigue at the question level, ranging from 4 of 25 (16%) for relatively simple tasks involving basic information retrieval (“What was the patient’s last outpatient weight prior to this ICU admission?”) to 15 of 25 (60%) for tasks involving clinical ambiguity (“Reconcile a possibly spurious lab value”). Fifteen participants (60%) experienced fatigue by the end of reviewing case 3.

### Cumulative Fatigue Over Time

**Figure 2.**
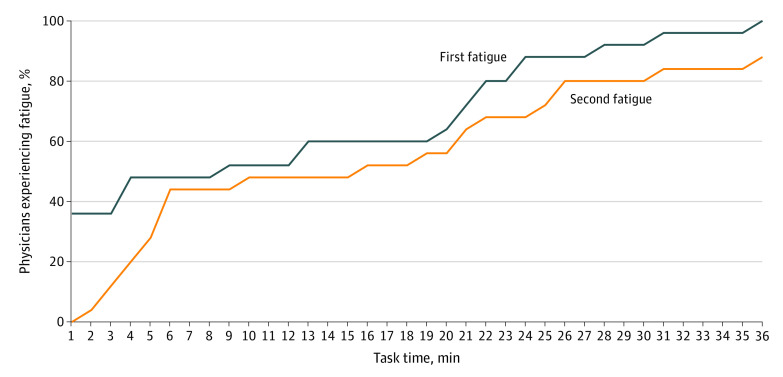
Cumulative Percentage of Users Experiencing Fatigue for the First and Second Instance During Electronic Health Record Simulation (N = 25)

Figure 2 shows the cumulative percentage of participants who experienced actual physiological fatigue at least once during the study, where each participant is counted as experiencing fatigue from the first instance. A total of 9 of 25 participants (36%) experienced fatigue within the first minute of the study; 16 of 25 participants (64%) experienced fatigue at least once within the first 20 minutes of the study, and 20 of 25 participants (80%) experienced fatigue after 22 minutes of EHR use. A sensitivity analysis was performed, in which we counted the second instance an individual experienced fatigue, and findings remained robust as 19 of 25 participants (76%) experienced a second instance of fatigue within 1 minute of the first instance (Figure 2).

**Figure 3.**
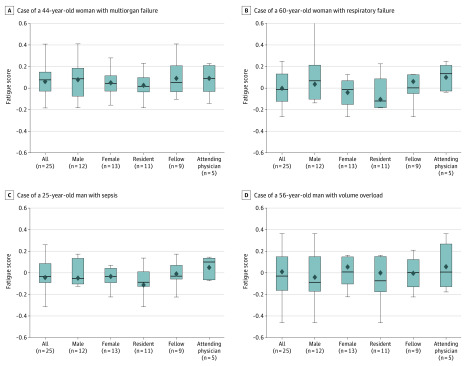
Distribution of Physician Fatigue Scores During Electronic Health Record Activity by Sex and Role A, Case of a 44-year-old woman with multiorgan failure. B, Case of a 60-year-old woman with respiratory failure. C, Case of a 25-year-old man with sepsis. D, Case of a 56-year-old man with volume overload. Lower fatigue scores indicate greater fatigue. The top and bottom bars indicate the first and third quartile, respectively; the diamond indicates the mean; the horizontal line in the bars indicate the median; and vertical lines indicate minimum and maximum values.

Figure 3 shows the distribution of physician fatigue scores at the case level, stratified by sex and clinical role. Across all participants, mean fatigue scores remained similar from 1 case to the next and tightly clustered around 0; however, we did see some variation. Overall fatigue scores were negative for cases 2 and 3. Although there were differences in mean scores across different subgroups, these differences were not statistically significantly different (Figure 3).

### Efficiency

Participants completed the study in a mean (SD) of 34:43 (11:41) minutes, using 304 (79) mouse clicks, and visiting 85 (19) EHR screens (Table). Female physicians were faster than male physicians (mean [SD], 31:37 [8:22] vs 38:04 [13:40] minutes) but required more mouse clicks (mean [SD], 355 [101] vs 301 [66]). Fellows were faster (mean [SD], 28:51 [5:52] vs 36:54 [14:43] minutes) and more efficient (mean [SD], 312.7 [88] vs 411.6 [90] mouse clicks) compared with residents. Attending physicians visited the fewest EHR screens compared with fellows and residents (mean [SD], 73 [8] vs 81 [16] vs 94 [21]). None of the observed sex- or role-based differences in EHR efficiency reached statistical significance. One participant spent noticeably more time than the mean on the simulation task (approximately 73 minutes compared with a mean of approximately 34 minutes). Sensitivity analyses conducted with the omission of this participant led to no significant differences in study findings.

### The Carryover Association of EHR-Related Fatigue With Physician Efficiency

**Figure 4.**
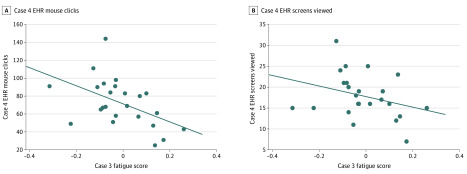
Association Between Fatigue Score in 1 Case and Electronic Health Record (EHR) Efficiency in the Subsequent Case A, Fatigue score in case 3 and efficiency (number of mouse clicks) in case 4. B, Fatigue score in case 3 and efficiency (number of EHR screens viewed) in case 4.

Physicians’ EHR efficiency was negatively associated with having experienced EHR-related fatigue. We observed a pattern in physicians’ EHR use after experiencing fatigue in 1 case such that the subsequent case required more time, mouse clicks, and EHR screen visits to complete, irrespective of the nature or order of the case. These results suggest a carryover association: when participants experienced greater fatigue during 1 patient case (as evidenced by more negative fatigue scores), they were less efficient using the EHR during the subsequent patient case. Figure 4A and B provide scatterplots mapping these associations.

Significant negative correlations were found between: fatigue scores for case 2 and the number of mouse clicks in case 3 (*r* = −0.481; *P* = .01), fatigue scores for case 3 and the number of mouse clicks in case 4 (*r* = −0.562; *P* = .003), fatigue scores in case 3 and the time to complete case 4 (*r* = −0.521; *P* = .007), and fatigue scores in case 3 and the number of EHR screens visited in case 4 (*r* = −0.486; *P* = .01). The association between fatigue scores for case 1 and the number of EHR screens visited in case 2 was not significant (*r* = −0.381; *P* = .06).

Our sensitivity analysis of the carryover showed similar patterns. When removing outliers, we observed the same negative correlations between fatigue scores and efficiency measures in the subsequent cases, as shown in Figure 4 and eFigure 2 and eTable 3 in the Supplement.

## Discussion

To our knowledge, this cross-sectional, simulation-based EHR usability study is the first to use pupillometry to assess the association of EHR activity with fatigue and efficiency among ICU physicians. We report that 20 of 25 physician participants (80%) experienced physiological fatigue at least once in 22 minutes of EHR use, as measured by pupillometry. Experiencing EHR-related fatigue was negatively associated with EHR efficiency as measured by time, mouse clicks, and screen visits.

We observed a carryover association: when participants experienced greater fatigue during 1 patient case, they were less efficient using the EHR during the subsequent patient case. There was an inverse association and a temporal component between fatigue scores and multiple domains of EHR efficiency spanning patient cases. This finding was most consistent with mouse clicks: across multiple sets of consecutive cases, lower fatigue scores on 1 case (indicating greater physiological fatigue) were associated with more mouse clicks on the subsequent case. To a lesser degree, we also observed an association between greater physiological fatigue during 1 case and needing more time and more screen visits in the subsequent case, although this pattern was limited to just 1 set of consecutive patient cases. These findings are hypothesis-generating, especially from the standpoint of the patient: if clinicians experience EHR-induced fatigue during the care of 1 patient, it may be associated with the care of the next patient in ways that are worthy of further investigation.

When compared with a typical day in an ICU, the simulation undertested the clinical demands of a physician. First-year trainees routinely review 5 or more patients, while upper-level residents, fellows, and attending physicians routinely review 12 or more patients. Even small differences in EHR efficiency measures during a single patient case, such as 10 to 20 mouse clicks or 30 to 60 seconds, could be clinically significant to a busy physician when scaled to a typical workload of 12 or more patients. Thus, the preliminary findings of this study may be increasingly pronounced as the number of patients reviewed in the EHR rises.

### Previous Research Findings

Prior studies using pupillometry in EHR simulation have examined physician workload (pupil dilation) among emergency department and hospitalist physicians as well as physician workload (blink rates) among primary care physicians managing follow-up test results in the outpatient setting.^39,40,41,42^ Our study adds value by using pupillometry to characterize physician fatigue among intensivists managing critically ill patients, a particularly high-stakes setting. We also add nuance by extending our analysis to examine physician fatigue and EHR efficiency over time and across multiple cases, which mirrors the reality of clinical workflows in most inpatient settings.^27,43^ The finding that physiological fatigue appears to occur in short periods of EHR-related work among physicians is itself an important advancement, given that fatigue is one of the leading human factors associated with errors and accidents in the workplace^44,45^ and that it can co-occur with burnout.^46^

### Strengths and Limitations

This study has some strengths, including the use of high-fidelity patient cases and clinically relevant interactive tasks, inclusion of physicians from different levels of training and clinical experience, the use of a leading EHR system, and the relatively large sample size (n = 25) that exceeds the typical threshold for usability studies. Furthermore, our approach to identifying and quantifying fatigue is a conservative one because we use relative pupil size changes and baseline testing rather than instantaneous (absolute) changes, so our findings may understate the actual physiological burden of EHR-related fatigue.

There are limitations in the study methods, procedures, and analysis that could potentially lead to the misinterpretation of findings. First, as this was a single-site study, we cannot exclude the possibility of selection bias, although we aimed to achieve a balance of sex representation and clinical roles. Second, cases were not randomized between participants in the simulation task, so it is possible that the observed fatigue was associated with case order. We also did not control for case-level features such as clinical acuity or number of tasks that might have explained the differences in time, number of EHR screens, and mouse clicks. However, in the natural clinical environment, there will always be variation in case complexity and task requirements from one patient to the next, so we wanted to mimic clinical workflows in the real world. Third, because all participants used the same eye-tracking glasses, there is the possibility of nondifferential measurement bias in the pupillometry data, which would introduce a conservative bias. Fourth, we did not collect subjective measures of fatigue from participants, as doing so for each case and question would have interrupted the flow of the study. Thus, we are unable to analyze the moment-to-moment association between objective fatigue, which we report, and subjective fatigue, which may be more clinically relevant. Fifth, in one case, the eye-tracking built-in battery died, which required an interruption to the activity.

### Future Directions

These findings open the door for many potential research questions and opportunities for future work. Although we observed fatigue among participants using the EHR, it is unknown whether this fatigue was simply owing to the challenging nature of reviewing cases of critically ill patients or whether certain aspects of EHR design such as screen layouts or workflows played a role. Future research is needed to better understand the complex association between EHR-related fatigue and care outcomes. Additional work should randomize case order and should evaluate differences in perceived satisfaction and physiological fatigue levels since our preliminary findings may show a discrepancy in perceived and actual EHR association. Furthermore, testing should be expanded to include clinical practitioners from other roles whose work is EHR-intensive such as nursing, respiratory therapy, and social work. Finally, additional work is needed to better understand the association of user-centered design with EHR performance, satisfaction, usability, and patient outcomes.

## Conclusions

We observed high rates of fatigue among ICU physicians during short periods of EHR simulation, which was negatively associated with EHR efficiency and included a carryover association across patient cases. More research is needed to investigate the underlying causes of EHR-associated fatigue, to support user-centered EHR design, and to inform safe EHR use policies and guidelines.

## Notes

Supplement. **eFigure 1.** Eye-Tracking Device Used in the Study (Tobii Pro 2 Glasses) **eTable 1.** Description and Categorization of Each of the Four Patient Cases as Reported by MICU Domain Expert **eTable 2.** Individual Fatigue Scores for Each Eye and Total Fatigue Score for Each Participant, Averaged Across all Four Simulation Patient Cases **eFigure 2.** Scatter Plots of Carryover Effect Between Cases 3 and 4 Including Outliers **eTable 3.** Analysis With and Without Outliers
