# Errors in Demographic Data in Results Section of Abstract and Text and in the Table

**DOI:** 10.1001/jamanetworkopen.2020.13153

**Published:** 2020-06-24

In the Original Investigation titled “Association of Electronic Health Record Use With Physician Fatigue and Efficiency,”^1^ published online June 9, 2020, in the Results section of the Abstract and text and in the Table, the mean (SD) age of the 25 intensive care unit (ICU) physicians was 33.2 (6.1) years. Also in the Table, their mean level of experience with the electronic health record (EHR) system was 4.2 (1.3) years, and they spent a mean (SD) of 32.6 (23.0) hours per week using it. Among the 13 ICU physicians who were female, the mean (SD) age was 31.5 (3.1) years, the mean (SD) level of experience with the EHR system was 4.0 (1.0) years, and the mean (SD) duration of time spent using it was 35.3 (25.4) hours per week. Among the 12 ICU physicians who were male, the mean (SD) age was 34.9 (7.6) years, the mean (SD) level of experience with the EHR system was 4.3 (1.4) years, and the mean (SD) duration of time spent using it was 29.8 (17.9) hours per week. These errors and their subsequent correction do not change the findings of the article. This article has been corrected.
