# Supplementary material for: Association of Electronic Health Record Use With Physician Fatigue and Efficiency
Source: JAMA Netw Open. 2020 Jun 9;3(6):e207385. doi: 10.1001/jamanetworkopen.2020.7385 (PMC7284310; doi:10.1001/jamanetworkopen.2020.7385)
Supplement: Supplement. — eFigure 1. Eye-Tracking Device Used in the Study (Tobii Pro 2 Glasses) eTable 1. Description and Categorization of Each of the Four Patient Cases as Reported by MICU Domain Expert eTable 2. Individual Fatigue Scores for Each Eye and Total Fatigue Score for Each Participant, Averaged Across all Four Simulation Patient Cases eFigure 2. Scatter Plots of Carryover Effect Between Cases 3 and 4 Including Outliers eTable 3. Analysis With and Without Outliers [file jamanetwopen-3-e207385-s001.pdf]

Supplementary Online Content

Khairat S, Coleman C, Ottmar P, Jayachander DI, Bice T, Carson SS. Association of electronic health record use with physician fatigue and efficiency. *JAMA Netw Open.* 2020;3(6):e207385. doi:10.1001/jamanetworkopen.2020.7385

eFigure 1. Eye-Tracking Device Used in the Study (Tobii Pro 2 Glasses)
eTable 1. Description and Categorization of Each of the Four Patient Cases as Reported by MICU Domain Expert
eTable 2. Individual Fatigue Scores for Each Eye and Total Fatigue Score for Each Participant, Averaged Across all Four Simulation Patient Cases
eFigure 2. Scatter Plots of Carryover Effect Between Cases 3 and 4 Including Outliers
eTable 3. Analysis With and Without Outliers

This supplementary material has been provided by the authors to give readers additional information about their work.

© 2020 Khairat S et al. *JAMA Network Open.*

eFigure 1. Eye-Tracking Device Used in the Study (Tobii Pro 2 Glasses)

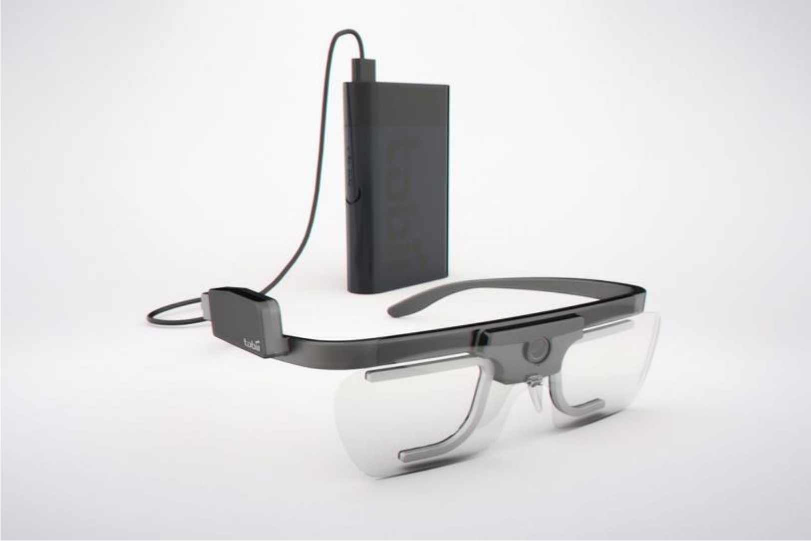

© 2020 Khairat S et al. *JAMA Network Open.*

eTable 1. Description and Categorization of Each of the Four Patient Cases as Reported by MICU Domain Expert

Case Description	Interactive Questions and Tasks
**1. Multi-system Organ Failure**<br>44 year-old female with multisystem organ failure and undifferentiated shock.<br><br>*Participants review clinical documentation, manage medications, and respond to consultations*	• How many services have been consulted?<br>• How many consult teams have seen the patient?<br>• Have labs been ordered per ID consultant?<br>• Have labs been collected?<br>• Do current abx, as ordered, match plan in note?<br>• Correct order modification?
**2. Acute Hypoxic Respiratory Failure**<br>60 year-old female with acute hypoxic respiratory failure due to pneumonia.<br><br>*Participants review clinical documentation and flowsheets, evaluate changes in mechanical ventilation, and analyze microbiology data*	• Was there a change in ventilator settings?<br>• What change(s) occurred?<br>• Why did change occur (clinical reason)?<br>• Are microbiology data available?<br>• Specific microbiology results?
**3. Sepsis**<br>25 year-old male with severe infection (sepsis) due to skin/soft tissue wound of the leg.<br><br>*Participants assess flowsheets, laboratory data, antibiotics and fluid management*	• Explanation for duplicate labs?<br>• Abx received since yesterday?<br>• Abx currently ordered?<br>• IV fluids received since yesterday?<br>• IVF administered = clinically appropriate?<br>• Are any labs currently ordered?<br>• Order additional lab tests
**4. Volume Overload**<br>56 year-old male trauma patient with postoperative heart failure and volume overload.<br><br>*Participants identify weight trends during previous visits, manage IV fluids and medications*	• Net fluid status since admission?<br>• Current weight?<br>• Last clinic weight?<br>• Manage IVF orders

© 2020 Khairat S et al. *JAMA Network Open.*

eTable 2. Individual Fatigue Scores for Each Eye and Total Fatigue Score for Each Participant, Averaged Across all Four Simulation Patient Cases

Participant	Right eye fatigue score	Left eye fatigue score	Total Fatigue Score
**P1**	0.324	0.206	0.265
**P2**	0.351	-0.121	0.115
**P3**	-0.579	0.265	-0.157
**P4**	-0.517	-0.327	-0.422
**P5**	-0.089	-0.196	-0.1425
**P6**	-0.107	-0.281	-0.194
**P7**	-0.2	-0.341	-0.2705
**P8**	-0.057	-0.064	-0.0605
**P9**	-0.113	-0.345	-0.229
**P10**	-0.189	-0.313	-0.251
**P11**	-0.026	0.025	-0.0005
**P12**	0.111	0.06	0.0855
**P13**	-0.074	-0.08	-0.077
**P14**	-0.054	-0.069	-0.0615
**P15**	-0.2	-0.272	-0.236
**P16**	-0.145	-0.161	-0.153
**P17**	-0.828	-0.901	-0.8645
**P18**	-0.105	-0.069	-0.087
**P19**	-0.174	0.49	0.158
**P20**	0.324	0.308	0.316
**P21**	-0.146	-0.113	-0.1295
**P22**	-0.303	-0.31	-0.3065
**P23**	0.056	0.03	0.043
**P24**	-0.377	-0.358	-0.3675
**P25**	-0.256	-0.284	-0.27

© 2020 Khairat S et al. *JAMA Network Open.*

eFigure 2. Scatter Plots of Carryover Effect Between Cases 3 and 4 Including Outliers

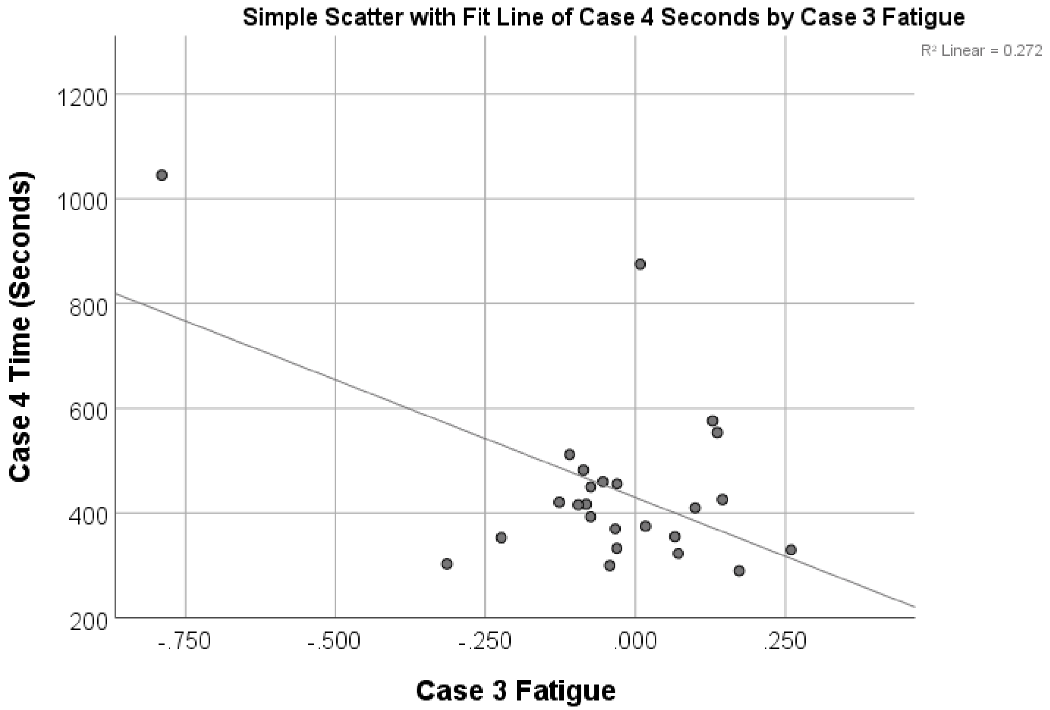

© 2020 Khairat S et al. *JAMA Network Open.*

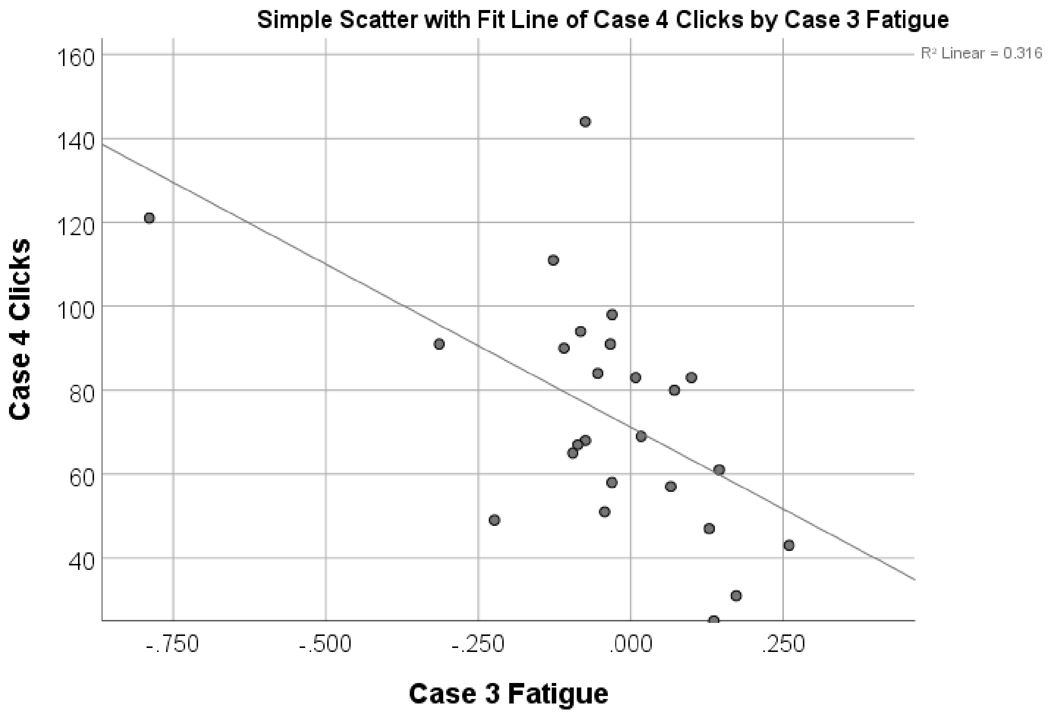

© 2020 Khairat S et al. *JAMA Network Open.*

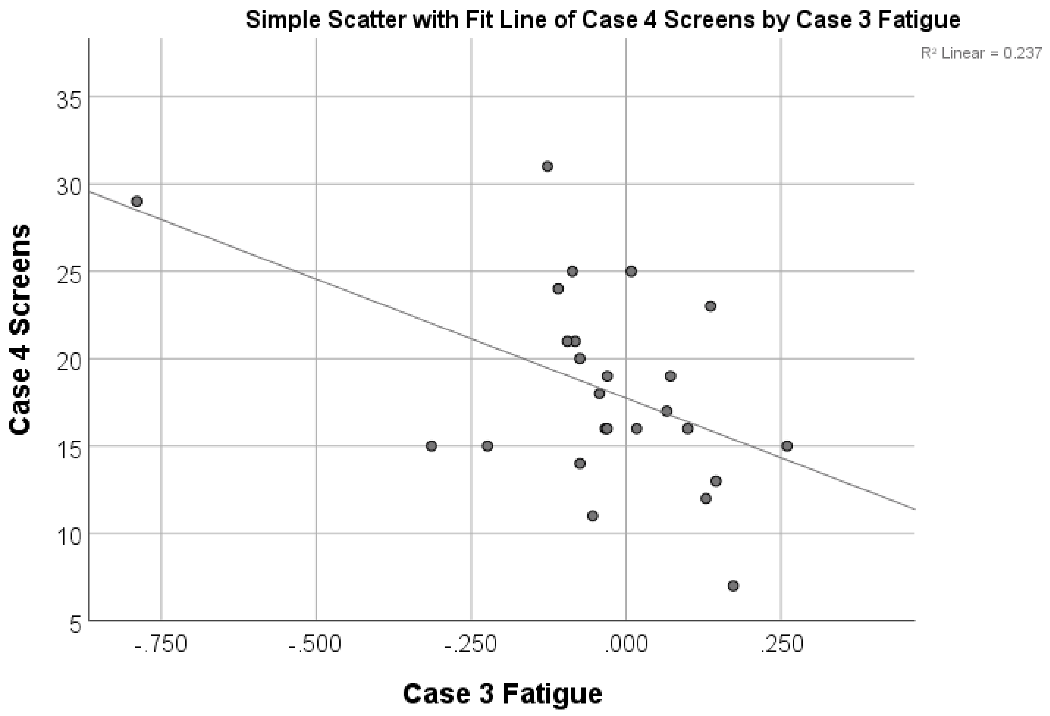

© 2020 Khairat S et al. *JAMA Network Open.*

eTable 3. Analysis With and Without Outliers

Analysis With Outliers

Case 1 Fatigue -> Case 2 Efficiency

Pearson Correlation Coefficients, N = 25<br>Prob > |r| under H0: Rho=0					
	**fatigue**	**screens**	**clicks**	**minutes**	**seconds**
**fatigue**	1.00000	-0.38060<br>0.0605	-0.26020<br>0.2091	0.15583<br>0.4570	0.15583<br>0.4570
**screens**	-0.38060<br>0.0605	1.00000	0.32554<br>0.1123	-0.04419<br>0.8339	-0.04419<br>0.8339
**clicks**	-0.26020<br>0.2091	0.32554<br>0.1123	1.00000	0.26871<br>0.1940	0.26871<br>0.1940
**minutes**	0.15583<br>0.4570	-0.04419<br>0.8339	0.26871<br>0.1940	1.00000	1.00000<br><.0001
**seconds**	0.15583<br>0.4570	-0.04419<br>0.8339	0.26871<br>0.1940	1.00000<br><.0001	1.00000

© 2020 Khairat S et al. *JAMA Network Open.*

Case 2 Fatigue -> Case 3 Efficiency

Pearson Correlation Coefficients, N = 25<br>Prob > |r| under H0: Rho=0					
	**fatigue**	**screens**	**clicks**	**minutes**	**seconds**
**fatigue**	1.00000	-0.04383<br>0.8352	-0.48106<br>0.0149	-0.22906<br>0.2707	-0.22906<br>0.2707
**screens**	-0.04383<br>0.8352	1.00000	0.39073<br>0.0535	-0.14633<br>0.4852	-0.14633<br>0.4852
**clicks**	-0.48106<br>0.0149	0.39073<br>0.0535	1.00000	0.43487<br>0.0298	0.43487<br>0.0298
**minutes**	-0.22906<br>0.2707	-0.14633<br>0.4852	0.43487<br>0.0298	1.00000	1.00000<br><.0001
**seconds**	-0.22906<br>0.2707	-0.14633<br>0.4852	0.43487<br>0.0298	1.00000<br><.0001	1.00000

© 2020 Khairat S et al. *JAMA Network Open.*

Case 3 Fatigue -> Case 4 Efficiency

Pearson Correlation Coefficients, N = 25
Prob > |r| under H0: Rho=0

	fatigue	screens	clicks	minutes	seconds
**fatigue**	1.00000	-0.48640<br>0.0137	-0.56229<br>0.0034	-0.52154<br>0.0075	-0.52154<br>0.0075
**screens**	-0.48640<br>0.0137	1.00000	0.48932<br>0.0130	0.54252<br>0.0051	0.54252<br>0.0051
**clicks**	-0.56229<br>0.0034	0.48932<br>0.0130	1.00000	0.29902<br>0.1465	0.29902<br>0.1465
**minutes**	-0.52154<br>0.0075	0.54252<br>0.0051	0.29902<br>0.1465	1.00000	1.00000<br><.0001
**seconds**	-0.52154<br>0.0075	0.54252<br>0.0051	0.29902<br>0.1465	1.00000<br><.0001	1.00000

© 2020 Khairat S et al. *JAMA Network Open.*

Analysis Without Outliers

Case 1 Fatigue and Case 2 efficiency

Pearson Correlation Coefficients, N = 24<br>Prob > |r| under H0: Rho=0					
	**fatigue**	**screens**	**clicks**	**minutes**	**seconds**
**fatigue**	1.00000	-0.31252<br>0.1371	-0.21873<br>0.3045	0.26717<br>0.2069	0.26717<br>0.2069
**screens**	-0.31252<br>0.1371	1.00000	0.30315<br>0.1499	-0.07976<br>0.7110	-0.07976<br>0.7110
**clicks**	-0.21873<br>0.3045	0.30315<br>0.1499	1.00000	0.25359<br>0.2318	0.25359<br>0.2318
**minutes**	0.26717<br>0.2069	-0.07976<br>0.7110	0.25359<br>0.2318	1.00000	1.00000<br><.0001
**seconds**	0.26717<br>0.2069	-0.07976<br>0.7110	0.25359<br>0.2318	1.00000<br><.0001	1.00000

© 2020 Khairat S et al. *JAMA Network Open.*

Case 2 fatigue and case 3 efficiency

Pearson Correlation Coefficients, N = 23<br>Prob > |r| under H0: Rho=0					
	**fatigue**	**screens**	**clicks**	**minutes**	**seconds**
**fatigue**	1.00000	-0.11835<br>0.5907	-0.01230<br>0.9556	0.58993<br>0.0030	0.58993<br>0.0030
**screens**	-0.11835<br>0.5907	1.00000	0.55494<br>0.0060	-0.10859<br>0.6219	-0.10859<br>0.6219
**clicks**	-0.01230<br>0.9556	0.55494<br>0.0060	1.00000	0.10587<br>0.6307	0.10587<br>0.6307
**minutes**	0.58993<br>0.0030	-0.10859<br>0.6219	0.10587<br>0.6307	1.00000	1.00000<br><.0001
**seconds**	0.58993<br>0.0030	-0.10859<br>0.6219	0.10587<br>0.6307	1.00000<br><.0001	1.00000

© 2020 Khairat S et al. *JAMA Network Open.*

Case 3 fatigue and case 4 efficiency

Pearson Correlation Coefficients, N = 24<br>Prob > |r| under H0: Rho=0					
	fatigue	screens	clicks	minutes	seconds
**fatigue**	1.00000	-0.31057<br>0.1397	-0.49279<br>0.0144	0.07615<br>0.7236	0.07615<br>0.7236
**screens**	-0.31057<br>0.1397	1.00000	0.40835<br>0.0476	0.40490<br>0.0497	0.40490<br>0.0497
**clicks**	-0.49279<br>0.0144	0.40835<br>0.0476	1.00000	0.07497<br>0.7277	0.07497<br>0.7277
**minutes**	0.07615<br>0.7236	0.40490<br>0.0497	0.07497<br>0.7277	1.00000	1.00000<br><.0001
**seconds**	0.07615<br>0.7236	0.40490<br>0.0497	0.07497<br>0.7277	1.00000<br><.0001	1.00000

© 2020 Khairat S et al. *JAMA Network Open.*
